# Bilateral Insufficiency Fracture of the Pelvis Following THA: A Case Report

**DOI:** 10.1155/2012/170736

**Published:** 2012-03-25

**Authors:** Shinya Hayashi, Takayuki Nishiyama, Takaaki Fujishiro, Shingo Hashimoto, Masahiro Kurosaka

**Affiliations:** Department of Orthopaedic Surgery, Graduate School of Medicine, Kobe University, 7-5-1 Kusunokicho Chuoku, Kobe 650-0017, Japan

## Abstract

Insufficiency fracture is of the stress fractures and is caused by repetitive stress on fragile bone. Insufficiency fractures of pubic rami are rare occurrences in association with total hip arthroplasty (THA). Postoperative stress fractures occur due to increase of patients activity following years of disability. The physician should consider the possibility of a pelvic insufficiency fracture in patients with RA after THA, if the patients present with groin pain. We demonstrate here the first case of bilateral insufficiency fracture of pubic rami and iliac bone following THA.

## 1. Introduction

THA is effective for decreasing pain and improving the loss of function. Acute hip pain within the first few years after THA is unusual when the components are correctly positioned. The differential diagnosis, dislocation, infection, and periarticular bursitis or tendinitis, are considered. Insufficiency fracture around components also should be considered as part of differential diagnosis.

Several reports showed cases of insufficiency fracture of pubic rami after THA [1–3]. However, insufficiency fracture of bilateral pubic rami and iliac bone following THA has never been reported. Here, we report a case of a patient who complained of bilateral pelvic pain following bilateral THA who was found to have bilateral insufficiency fractures of pubic rami and iliac bone.

## 2. Case Report

A 62-year-old woman suffering from rheumatoid arthritis (RA) underwent a left THA in 2007 and a right revision THA in 2009. The patient was treated with various doses of glucocorticoid for long term. After surgery, she was able to walk without support. However, ten months postoperatively, she presented with complaints of left groin pain that initially was associated with physical therapy sessions. The symptoms became more constant. She denied a history of a fall or of another traumatic event. Radiographs, at that time, did not show any significant findings. She was treated with anti-inflammatory medication and weight-bearing with use of a cane in the right hand. Over the next 2 months, the left groin pain was disappeared. After 4 month, the left pubic pain was recurrent. She did weight-bearing with use of a cane in the right hand again. However, she presented right groin pain several days later. Symptoms were worse with weight bearing and walking. Furthermore, several days later, both sides of buttock pain were presented and unable to walk.

**Figure 1:**
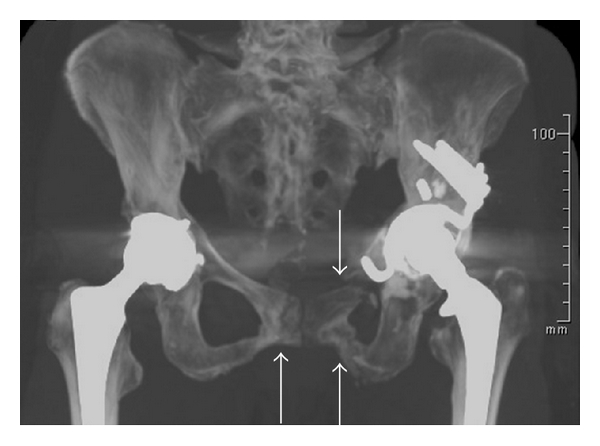
CT image showing pelvis. Arrows indicate fracture site of pubic rami.

**Figure 2:**
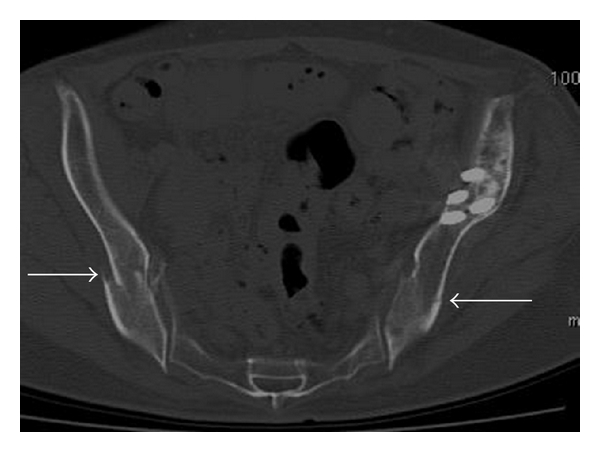
CT image showing pelvis. Arrows indicate fracture site of iliac bone.

Radiographs showed fractures of both pubic rami. Computed tomography (CT) showed bilateral fracture of pubic rami and iliac bone (Figures 1 and 2). Evaluation for metabolic bone disease was performed. There was no significant evidence evaluated by blood examination.

She was admitted to our hospital and took bed rest for 1 month and then used walker for 2 month. By this time, all symptoms had subsided.

## 3. Discussion

Stress fractures are classified into three subgroups: fatigue fracture caused by repetitive stress for normal bone, insufficiency fracture caused by repetitive stress for fragile bone, and pathologic fracture caused by repetitive stress for fragile invaded bone by tumor [4]. Zwartelé et al. reported systematic reviews of cementless THA in RA patients, and they concluded that cementless acetabular cups were rarely associated with mechanical complications including acetabular fracture [5]. However, Fukunishi et al. reported that 11 patients in 171 RA patients (6.4%) who underwent cemented THA suffered from pubic rami fractures postoperatively [6]. Isdale reported the bone quality in RA patients was poor, and insufficiency fractures of the pubic rami in RA appear to be more common than had been recognized [7]. In our case, the subtype of stress fracture is thought to be classified into insufficiency fracture caused by repetitive stress for fragile bone due to RA. The bone quality of this patient might be poor, because the patient was treated with glucocorticoid for long term.

The patient performed revision THA by using Kerboull-type acetabular reinforcement device. Several reports demonstrated the results postoperatively using Kerboull-type acetabular reinforcement device [8, 9]. However, there was no report of pubic rami fracture after THA. Therefore, the fracture may not be associated with acetabular component design.

Most pelvic insufficiency fractures occur in women, particularly those with osteoporotic bone. The patients have impaired activity levels prior to THA. After surgery, many of these patients are pain free and get activity at a much higher level than preoperatively. Therefore, they are susceptible to the development of an insufficiency fracture if the bone quality is poor. In our case, the mechanism of fracture is thought as follows. Firstly, the insufficiency fracture of left pubic rami occurred due to daily activity. The patient was placed on limited weight-bearing, with use of a cane in the right hand. This increased the stress on the right side, and the fracture of right pubic rami was occurred. Both sides of pubic fracture increased the stress on both iliac bone, and the fractures of both iliac bones were caused.

Insufficiency fractures are differentiated from acute fractures, which occur early in the postoperative period as a result of press-fit insertion of cementless acetabular components in osteopenic bone [10].

The physician should consider the possibility of a pelvic insufficiency fracture in patients with RA after THA. If the patient presents with groin pain, the patient should be treated with weight-bearing as conservative management.

However, pelvic discontinuity by both pubic rami and iliac bone fracture occurred in our case; even we recommended weight-bearing at the time of initial fracture of left pubic rami. We reported the first case of bilateral insufficiency fracture of pubic rami and iliac bone following THA.
